# Catabolism of hyaluronan: involvement of transition metals

**DOI:** 10.2478/v10102-009-0026-y

**Published:** 2009-12-28

**Authors:** Ladislav Šoltés, Grigorij Kogan

**Affiliations:** 1 Institute of Experimental Pharmacology, Slovak Academy of Sciences, SK-84104 Bratislava, Slovakia; 2 Directorate Health, Directorate General Research, European Commission, B-1049, Brussels, Belgium

**Keywords:** hyaluronan catabolism, synovial fluid, joint, transition metals, peroxidation, oxidative stress

## Abstract

One of the very complex structures in the vertebrates is the joint. The main component of the joint is the synovial fluid with its high-molar-mass glycosaminoglycan hyaluronan, which turnover is approximately twelve hours. Since the synovial fluid does not contain any hyaluronidases, the fast hyaluronan catabolism is caused primarily by reductive-oxidative processes.

Eight transition metals – V^23^, Mn^25^, Fe^26^, Co^27^, Ni^28^, Cu^29^, Zn^30^, and Mo^42^ – naturally occurring in living organism are essential for the control of various metabolic and signaling pathways. They are also the key elements in catabolism of hyaluronan in the joint.

In this overview, the role of these metals in physiological and pathophysiological catabolism of hyaluronan is described. The participation of these metals in the initiation and propagation of the radical degradation hyaluronan is critically reviewed.

## Biogenic elements

Virtually each of the chemical elements plays some role in the Earth's living systems, however, only 24 elements account for the vast majority of material in these systems. These elements are divided into (**i**) six major biogenic elements, namely carbon, hydrogen, oxygen, nitrogen, sulfur, and phosphorus, (**ii**) five minor biogenic elements, sodium, potassium, magnesium, calcium, and chlorine, and (**iii**) thirteen biogenic trace elements comprising manganese, iron, cobalt, copper, zinc, boron, aluminum, vanadium, molybdenum, iodine, silicon, nickel, and bromine.

## Transition metals

Thirty eight chemical elements, with the atomic numbers 21 to 30, 39 to 48, 72 to 80, and 104 to 112, are termed transition metals (cf. Table 1). Of these the following eight – vanadium, manganese, iron, cobalt, nickel, copper, zinc, and molybdenum – belong to the biogenic trace elements. Thus V^23^, Mn^25^, Fe^26^, Co^27^, Ni^28^, Cu^29^, Zn^30^, and Mo^42^ could be classified as “biogenic transition metals”.

**Table 1:** Transition metals.

Group	3 (III B)	4 (IV B)	5 (V B)	6 (VI B)	7 (VII B)	8 (VIII B)	9 (VIII B)	10 (VIII B)	11 (I B)	12 (II B)
**Period 4**	Sc ^21^	Ti ^22^	V ^23^	Cr ^24^	Mn ^25^	Fe ^26^	Co ^27^	Ni ^28^	Cu ^29^	Zn ^30^
	2, 8	2, 8	2, 8	2, 8	2, 8	2, 8	2, 8	2, 8	2, 8	2, 8
	9, 2	10, 2	11, 2	13, 1	13, 2	14, 2	15, 2	16, 2	18, 1	18, 2
**Period 5**	Y ^39^	Zr ^40^	Nb ^41^	Mo ^42^	Tc ^43^	Ru ^44^	Rh ^45^	Pd ^46^	Ag ^47^	Cd ^48^
	2, 8, 18	2, 8, 18	2, 8, 18	2, 8, 18	2, 8, 18	2, 8, 18	2, 8, 18	2, 8, 18	2, 8, 18	2, 8, 18
	9, 2	10, 2	12, 1	13, 1	14, 1	15, 1	16, 1	18, 0	18, 1	18, 2
**Period 6**	^*^	Hf ^72^	Ta ^73^	W ^74^	Re ^75^	Os ^76^	Ir ^77^	Pt ^78^	Au ^79^	Hg ^80^
		2, 8, 18, 32	2, 8, 18, 32	2, 8, 18, 32	2, 8, 18, 32	2, 8, 18, 32	2, 8, 18, 32	2, 8, 18, 32	2, 8, 18, 32	2, 8, 18, 32
		10, 2	11, 2	12, 2	13, 2	14, 2	15, 2	17, 1	18, 1	18, 2
**Period 7**	^**^	Rf ^104^	Db ^105^	Sg ^106^	Bh ^107^	Hs ^108^	Mt ^109^	Ds ^110^	Rg ^111^	Uub ^112^
		2, 8, 18, 32, 32	2, 8, 18, 32, 32	2, 8, 18, 32, 32	2, 8, 18, 32, 32	2, 8, 18, 32, 32	2, 8, 18, 32, 32	2, 8, 18, 32, 32	2, 8, 18, 32, 32	2, 8, 18, 32, 32
		10, 2	11, 2	12, 2	13, 2	14, 2	15, 2	17, 1	18, 1	18, 2

^*^ La – lanthanide series;

^**^ Ac – actinide series

The transition metals (in shaded boxes) – vanadium, manganese, iron, cobalt, nickel, copper, zinc, and molybdenum – belong to biogenic trace elements. The numbers in the first line under the element symbol represent number of electrons in the innermost (closest to the nucleus) orbitals, while those in the second line correspond to the electrons in the valence/binding orbitals.

In most cases, the valence electrons of the transition metal, i.e. those electrons used in formation of the bonds with other elements, are present in two outermost orbitals. For this reason, transition metals may exist in several oxidation states (characterized by the oxidation numbers). For the biogenic transition metals, these states/numbers are listed in Table 2.

**Table 2:** Oxidation states (oxidation numbers) of the biogenic transition metals.

Element	Oxidation states/numbers		Electronegativity (Pauling scale)	Ionization energies: 1^st^; 2^nd^; 3^rd^ [kJ/mol]
Vanadium	V^a^	II, III, IV (I)^b^	1.63	650.9; 1414; 2830
Manganese	II	III, IV, VI, VII, (I, V)	1.55	717.3; 1509; 3248
Iron	III	II, V, (IV, VI)	1.83	762.5; 1561.9; 2957
Cobalt	II	III, (IV)	1.88	760.4; 1648; 3232
Nickel	II	III, (I, IV)	1.91	737.1; 1753; 3395
Copper	II	I, (III, IV)	1.90	745.5; 1957.9; 3555
Zinc	II		1.65	906.4; 1733.3; 3833
Molybdenum	VI	II, III, IV, V	2.16	684.3; 1560; 2618

^a,b^ The most (2^nd^ column) and the less (3^rd^ column) common oxidation states/numbers.

^b^ Rare oxidation states/numbers are given in parentheses.

Transition metals may form complexes with both charged and neutral ligands. A variety of different oxidation states as well as the coordination by ligands provide redox and/or catalytic activity to transition metals, which serve as the catalytic centers of enzymes (oxidases, dehydrogenases).

Most biogenic transition metals participate in the control of various metabolic and signaling pathways. However, their versatile coordination chemistry and redox properties allow them to escape the control mechanisms, such as homeostasis, transport, compartmentalization, and binding to the designated tissue and cell constituents (Valko *et al*., 2005).

When these metals are liberated from their catalytic sites within enzymes and sequestered abnormally by other ligands, the consequences can be deleterious for the environment. Under such circumstances, irreversible oxidative changes to both the metal-complexing ligand itself and the neighboring molecules that intercept the reactive intermediates of the initial reaction, may initiate a cascade of oxidative stress events (HaMai *et al*., 2001).

Moreover, transition metal ions have a specific feature, the so-called “isoelectronics”. The manganeous ions are electronically equivalent to the ferric ones, both having five electrons (*d* ^5^ configuration) in the valence subshells, which predetermine their mutual substitutability, e.g. in the octahedral coordination complexes. A similar isoelectronics, within tetrahedral coordination complexes, can be found for Zn(II) and Cu(I), i.e. both possess *d* ^10^ configuration, or for a less common pair Fe(II) and Co(III) – the *d* ^6^ configuration of the valence electrons. Such an isoelectronic effect may allow ions of two different elemental species to compete for the same ligands.

**Scheme 1:**
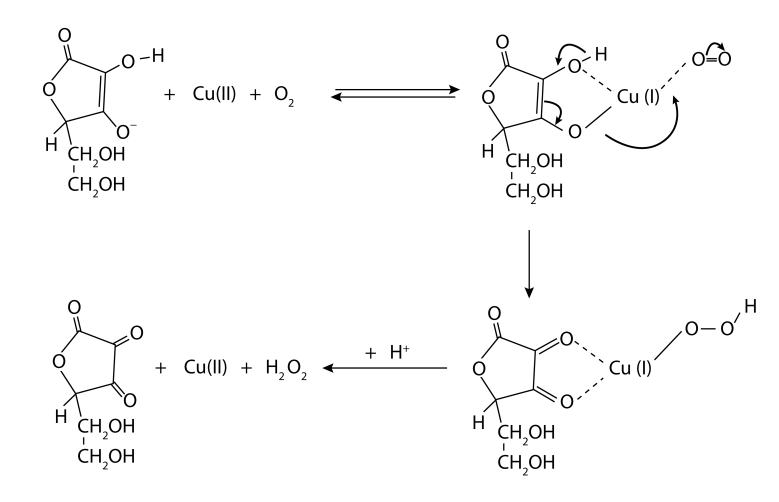
Generation of H_2_O_2_ by Weissberger's system from ascorbate and Cu(II) under aerobic conditions (adapted from Fisher and Naughton, 2005).

Coordination of iron with biomolecules involves the participation of its *d* orbitals. Since molecular oxygen using its electrons in antibonding π* orbitals can ligate to iron by overlapping its *d* orbitals, iron may serve as a bridge between the biomolecule and oxygen. The “flexibility” of iron not only means its ability to vary the oxidation state, but also implies its capacity to change electronic spin properties and relative redox potential in response to interaction with different coordinating ligands. When ascorbate is the coordinating biomolecule/ligand, it acts as both the iron chelator and reductant. Hence ascorbate upon binding with Fe(III) reduces iron to the Fe(II) ion, which forms a coordination complex ascorbate-Fe(II)-oxygen (Valko *et al*., 2005). This so-called Udenfriend's oxidative complex/system is a very efficient reagent, used by organic chemists, to hydroxylate aromatic compounds, saturate hydrocarbons to alcohols, olefins to epoxides, *etc*. (Udenfriend *et al*., 1954). With copper, the so-called Weissberger's system – ascorbate-Cu(I)-oxygen (Weissberger *et al*., 1943; Khan and Martell, 1967) – may generate hydrogen peroxide (cf. Scheme 1) (Fisher and Naughton, 2003; 2004; 2005). Due to H_2_O_2_ decomposition through Fenton-like reaction, the Weissberger's system belongs to the most efficient generators of hydroxyl radicals (Šoltés *et al*., 2006).

In the case when Udenfriend's or Weissberger's systems do not have additional oxidizable substrate, one speaks of “ascorbate auto-oxidation” (Buettner and Jurkiewicz, 1993), where the resulting reaction product is dehydroascorbate (DHA). However, in the simultaneous presence of oxidizable substrates, the reaction products comprise both oxidized/decomposed substrates (Šoltés *et al*., 2007).

### Vanadium

The oxidation states of vanadium include **+5**, +2, +3, and +4; that of +1 is rarely observed (Table 2). Vanadium is an essential element of some enzymes (e.g. nitrogenases in nitrogen-fixing micro-organisms). Rats and chickens are known to require vanadium; deficiency in vanadium results in their reduced growth and impaired reproduction. Administration of oxovanadium compounds was shown to alleviate *Diabetes mellitus* manifestations in certain animal models and humans.

### Manganese

The common oxidation states/numbers of manganese are **+2**, +3, +4, +6, and +7, although oxidation states +1 and +5 have also been observed (Table 2). Compounds, in which manganese exists in higher oxidation states are powerful oxidizing agents. Potassium permanganate (oxidation number +7) is used in medicine as an efficient disinfectant. Mn(III)-acetate is a relatively powerful oxidizing agent; the Mn(III)/Mn(II) standard reduction potential E’° equals +1.5 V.

Manganese ions function as cofactors for a number of enzymes, of which Mn-containing superoxide dismutase (Mn-SOD) is one of the most essential enzymes required for almost all organisms living in the presence of oxygen. They use Mn-SOD to deal with the toxic effects of superoxide anion radicals (O_2_ ·−), formed by the one-electron reduction of dioxygen. Exceptions include *Lactobacilli* bacteria, which for the task of O_2_ ·− detoxification use a different non-enzymatic mechanism involving Mn(II) complexed directly with polyphosphate. Further classes of enzymes, which may have manganese “cofactors”, include oxidoreductases, transferases, hydrolases, lyases, isomerases, and ligases.

The human body contains about 300 ppm of manganese; the recommended daily intake of this essential element is 3–9 mg (Roth, 2006). The low manganese turnover observed in patients with rheumatoid arthritis may cause disturbances in the synthesis of mucopolysaccharides observed in experimental animals with manganese deficiency (Niedermeier and Griggs, 1971). A heavy exposure to manganese can, however, cause chronic manganism – a form of Parkinson's disease.

Manganous ions are paramagnetic and hence they are detectable by EPR spectroscopy. Six-band EPR spectrum of Mn(II) has been detected in most natural products.

### Iron

The Fe(III)/Fe(II) reduction potential at pH 7 equaling +0.48 V (Koppenol, 1994) predetermines iron as one of the major candidates in the production (and metabolism) of free radicals in biological systems. Contrary to ferric salts, which at neutral pH precipitate forming oxyhydroxide aggregates, ferrous compounds are soluble, though unstable, since they tend to react with dioxygen yielding O_2_ ·− and Fe(III).

In mammals, the iron distribution is heavily regulated because Fe(II) ions represent a risk/toxic factor. Excessive iron intake can result in iron overload disorders, such as hemochromatosis, while diminished absorption of this essential biogenic element from the duodenum or deficit of this metal in food may lead to anemia.

The average-weight human body contains approximately 4–5 g of iron firmly bound/complexed in metalloproteins such as the oxygen carrier - hemoglobin, the enzymes containing heme prosthetic groups - catalase, cytochromes, and in the iron transport/storage proteins - transferrin, ferritin, and hemosiderin.

Transferrin, a glycoprotein, has two separate binding sites to which Fe(III) attaches extremely tightly. Under physiological conditions, the transport protein – transferrin – present in the bloodstream is, however, loaded only to ca. 30% of its total iron-binding capacity, so that the amount of “free” iron salts available in plasma should be negligible.

Ferritin is a high-capacity and low-affinity storage protein with the capacity of about 4,500 atoms of iron per one protein macromolecule. Iron from ferritin can be removed by the action of some “bioreductants”, such as ascorbate. However, in tissues only trace amounts of iron are thought to remain “free” (non-chelated) or loosely chelated. Evidence was provided that iron may be present in tissues in the micromolar range complexed/bound to ATP, AMP, GTP, and possibly citrate (Koppenol, 1994).

### Cobalt

Cobalt is essential to humans. This metal ion is the central component of cobalamin (Vitamin B_12_).

### Nickel

The most common oxidation state of nickel is **+2**, although in Ni-complexes oxidation states +1, +3, and +4 have also been observed. Solubilized Ni(II) ions in aqueous media at physiological pH are hydrated to a hexahydrate [Ni(H_2_O)_6_]^2+^ cation.

The main Ni-transporting protein in blood is albumin, though nickeloplasmin also transports these metal ions. The role of nickel in enzymes such as urease, glyoxalase, and a class of superoxide dismutase has recently been recognized (Thornalley, 2003; Szilagyi *et al*., 2004).

The daily intake of nickel is estimated to be in the range 35–300 µg.

### Copper

An average adult human body contains about 80 mg of copper; the recommended copper intake for adults is 0.9 mg/day. When copper is first absorbed in the gut, it is transported to the liver tightly bound to albumin.

Copper is stored in hepatocytes bound to metallothionein. In the bloodstream, copper is bound/carried within a plasma protein – ceruloplasmin. Each ceruloplasmin macromolecule, which molar mass is around 134 kDa, complexes/binds up to eight copper ions, of which two can liberate relatively easily (Shukla *et al*., 2006). Redox-active copper is the principal constituent in a variety of enzymes, including cytochrome *c* oxidase and CuZn-superoxide dismutase (CuZn-SOD).

Copper toxicity is derived from its ability to accept and donate single electrons. It has been claimed that cuprous/cupric ions catalyze the production of hydroxyl radical in a manner analogous to Fenton chemistry. This increase in unmediated reactive radicals, generally termed oxidative stress, is an active area of research in a variety of diseases where copper may play an insidious role.

In high amounts, copper can be poisonous and even fatal to organisms. This metal has been implicated in the pathogenesis of neurodegenerative disorders, such as Alzheimer's and Parkinson's diseases, as well as amyotrophic lateral sclerosis. In Wilson's disease, body tissues, mostly the liver and brain, retain too much copper. D-Penicillamine and oral Zn-supplements have been used for treating patients with Wilson's disease (Gaetke and Chow, 2003).

### Zinc

Zinc is a component of many metalloproteins such as metalloenzymes and metallothionein proteins. Chelatable redox-inert Zn(II) may serve as an antioxidant by preventing binding of pro-oxidant cuprous ions at tissue sites.

The adult human body contains approximately 1.5–2.5 g of zinc. The recommended dietary allowance of zinc is 11 mg for males and 8 mg for females, with higher amounts recommended during pregnancy and lactation. Brain development is stunted by zinc insufficiency *in utero* and in youth.

Zinc is excreted mostly in feces (12–15 mg/day) and lesser amounts (0.5 mg/day) are eliminated in urine.

### Molybdenum

Molybdenum is present in several enzymes such as aldehyde and sulfite oxidases. The purine catabolism, involving the oxidation of xanthine to uric acid, is catalyzed by xanthine oxidase – a Mo-containing enzyme.

A 70-kg human body contains about 10 mg of molybdenum, which occurs in higher concentrations in the liver and kidneys. The optimal daily intake of molybdenum is 0.3 mg. Molybdenum deficiency may cause growth retardation and impaired reproduction. High amounts of molybdenum can interfere with the body's uptake of copper.

## Non-enzymatic peroxidation – a radical chain reaction

As can be concluded from the above facts, biogenic transition-metal ions are sequestered by proteins: iron in transferrin and ferritin, copper in ceruloplasmin, *etc*. However, a small amount of low-molar-mass metal chelates/complexes is likely to be present at all times due to the transfer of metals from storage proteins to metalloproteins and the turnover of these proteins (Koppenol, 1994; Halliwell and Gutteridge, 1990).

Metal ions – especially those of iron – are capable of redox cycling by accepting [Fe(III) + 1e^–^ → Fe(II)] or donating [Fe(II) – 1e^–^ → Fe(III)] electron(s). These capabilities are closely related with the catalytic participation of iron in reactions producing oxygen-derived reactive species.

In a wide variety of *in vitro* systems, Fe(II) salts and/or non-enzyme chelated/complexed ferrous cations (e.g. Fe(II)-EDTA) were shown to enhance radical damage by increasing the production of oxygen-derived reactive species.

### Initiation reaction(s)

In a completely peroxide-free system, the initiation of a peroxidation sequence refers to the attack of any species that has sufficient reactivity to abstract a hydrogen atom (H· radical) from the (bio)substrate involved in a radical chain reaction. O_2_ ·− is insufficiently reactive to abstract the H· radical. The protonated form of O_2_ ·−, i.e. HO_2_ ·, although present in a trace amount, is more reactive and appears to be capable of abstracting the H· radical (Halliwell and Gutteridge, 1990). Alternatively, complexes of Fe(II) ions and dioxygen are also assumed to yield reactive species of unknown nature, which are subsequently able to oxidize biological material (Qian and Buettner, 1999; Flemmig and Arnhold, 2007). However, as generally believed, the most efficient acceptor of a H· radical should be the hydroxyl free radical – ·OH, which redox potential (·OH/H_2_O) equals +2.31 V at pH 7. For generation of ·OH radical(s), a sequence of the following reactions has been usually suggested

(i) $$\text{2Fe(II)+2}{\text{O}}_{\text{2}}\text{↔ 2Fe(III)+2}{\text{O}}_{\text{2}}^{\mathrm{\cdot -}}\text{(redox reaction; reversible)}$$

(ii) $$\text{2}{\text{O}}_{\text{2}}^{\mathrm{\cdot -}}\text{+2}{\text{H}}^{\text{+}}\text{→}{\text{H}}_{\text{2}}{\text{O}}_{\text{2}}\text{+}{\text{O}}_{\text{2}}\text{(dismutation reaction)}$$

$$\text{2Fe(II)+}{\text{O}}_{2}\text{+2}{\text{H}}^{+}\text{→2Fe(III)+}{\text{H}}_{2}{\text{O}}_{2}\text{net reaction}$$

(1) $$\text{Fe(II)+}{\text{H}}_{2}{\text{O}}_{2}\text{→Fe(III)+·OH + H}{\text{O}}^{\text{−}}\text{(Fenton reaction)}$$

(2) $${\text{O}}_{\text{2}}^{\mathrm{\cdot -}}\text{+}{\text{H}}_{\text{2}}{\text{O}}_{\text{2}}\text{↔ H}{\text{O}}^{\cdot }\text{+ H}{\text{O}}^{\text{−}}\text{+}{\text{O}}_{\text{2}}\text{(Haber-Weiss reaction; reversible)}$$

(It is, however, important to note that high Fe(II) concentration can diminish the overall yield of ·OH radicals by scavenging them [·OH Fe(II) → Fe(III) + HO^–^].)

As evident, both reactions **1** and **2** yield ·OH radicals, which may initiate e.g. peroxidation of (polyunsaturated) fatty acids in lipids (LH) and thus generate peroxyl lipid radicals (LOO·) by a sequence of the following reactions

(3) $$\text{LH+}\cdot \text{OH→L}\cdot \text{+}{\text{H}}_{\text{2}}\text{O}$$

(4) $$\text{L}\cdot \text{+}{\text{O}}_{\text{2}}\text{→ LO}\text{O}\cdot$$

### Propagation reaction(s)

The LOO· radicals propagate the lipid peroxidation chain reactions as follows

(5) LOO·+ LH→ LOOH +L·

and the reaction **5** is then followed by reaction **4**.

It should be pointed out that if a sufficiently high excess of ferrous cations is still present in the system, the produced lipid hydroperoxides (may) undergo an iron(II)-driven “decomposition” reaction

(6) $$\text{LOOH + Fe(II)→ L}\text{O}\cdot \text{+ H}{\text{O}}^{\text{−}}\text{+ Fe(III)}$$

yielding an alkoxyl lipid radical (LO·), which, due to its qualitatively similar reactivity as that of the lipid peroxyl radical (LOO·), may propagate the lipid peroxidation as follows

(7) LO·+ LH →LOH +L·

and the reaction **7** is then also followed by reaction **4**.

It has to be admitted that kinetically the reaction of Fe(II) with lipid hydroperoxides (reaction **6**) is very plausible to occur since it is by one order of magnitude faster than the Fe(II)-mediated reaction of H_2_O_2_ decomposition (the rate constant for reaction **1** (and reaction **2** as well) is about 76 M^−1^ s^−1^, while for reaction **6** the constant is equal to ca. 10^3^ M^−1^ s^−1^; Halliwell and Gutteridge, 1990).

To elucidate the frequently observed fact that the *in vitro* lipid peroxidation can be initiated also by ferric salts/complexes, it should be taken into account that during storage and handling under oxygen-containing atmosphere, all commercially available lipids are readily oxidized, yielding a “contaminant” – LOOH. And this is exactly the substance, which acts as an initiator of the (*in vitro*) lipid peroxidation due to the reaction

(8) $$\text{LOOH + Fe(III) → LO}\text{O}\cdot \text{+ Fe(II)+}{\text{H}}^{\text{+}}$$

that is subsequently followed by reaction **5**.

An important fact should be noted, namely that due to a high tendency of ferrous ions to be oxidized (the Fe(III)/Fe(II) reduction potential is equal to +0.48 V at pH 7 (Koppenol, 1994)), practically none of the Fe(II) salts/complexes is free of Fe(III) cations. Although Fe(III) should not trigger lipid peroxidation, due to ubiquitous contamination of lipids with the hydroperoxides LOOH, addition of Fe(III) initiates a radical chain reaction since its reaction with hydroperoxides generates LOO· radicals according to the reaction **8**.

If the potential of the Fenton reaction to take place is not unambiguously confirmed, one can come to a false conclusion that upon reacting with hydrogen peroxide any (biogenic) transition metal in lower oxidation state (M^n+^) would generate hydroxyl radicals by the reaction M^n+^ + H_2_O_2_ → M^(n+1)^+ ·OH + HO^–^. However, most researchers agree with the statement that under normal/physiological conditions *in vivo* only the iron(II)-dependent formation of ·OH radicals may actually occur (Halliwell and Gutteridge, 1990).

## Joint – role of hyaluronan

A joint is formed by the ends of two (or more) bones connected by connective tissues. The function of joints in the human organism is to ensure mutual motion of the adjacent bones in plane (bending *x* ↔ *y*) as well as in space (rotation *x* ↔ *y* ↔ *z*). The bone ends linked in a joint are covered with a thin soft layer – the cartilage, which matrix is being permanently restructured/rebuilt by the embedded chondrocytes.

Every joint is surrounded by a fibrous tissue capsule called synovium. The primary function of synovium is the production of synovial fluid (SF), which reduces friction and wear and tear of the joint. The main components of this “lubricating” fluid are a filtrate of blood plasma and a high-molar-mass polysaccharide – hyaluronan (HA), extruded by the type II synoviocytes, the cells localized within synovial membrane.

**Figure 1:**
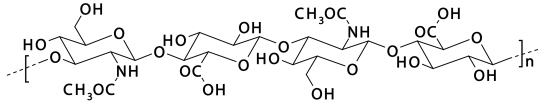
Hyaluronan – the acid form.

HA (Figure 1) is a linear high-molar-mass natural polysaccharide formed from disaccharide units containing *N*-acetyl-D-glucosamine and D-glucuronic acid. The chemical structure of HA is rather regular, the only deviation being a possible replacement of *N*-acetyl-D-glucosamine by deacetylated glucosamine residues. In an aqueous milieu, HA is represented by negatively charged hyaluronate macromolecules with extended conformations, which impart high viscosity/viscoelasticity to its solution (Hardingham, 2004).

In human beings, HA is abundantly present in almost all body fluids and tissues. In synovial fluid and vitreous humor, HA macromolecules reaching megadalton molar masses, are present in a “free form”, i.e. not associated with proteins. In the articular cartilage matrix, however, HA is associated via a link protein with a proteoglycan – aggrecan – consisting of collagen and glycosaminoglycans, namely chondroitin sulfate and keratan sulfate.

In a healthy human being, the synovial fluid containing along with the blood plasma filtrate also the entangled macromolecules of HA (1.4–3.6 mg/mL; Kogan *et al*., 2007), represents a gel-like colloidal medium. The nutrients, including oxygen supply, upon crossing the synovial barrier permeate through the viscous colloid to the avascular articular cartilage where they are utilized by embedded chondrocytes. On the other hand, the chondrocyte catabolites (should) cross both the viscous fluid and synovial membrane. It can thus be concluded that within the articular gel-like fluid, the process of “mixing” at the increased mobility of the joint, may significantly affect joint homeostasis.

## Hypoxia and re-oxygenation of the joint

Taking into consideration that cartilage does not contain any teleneurons, regulation of chondrocytes should be of chemical nature. In the relaxed state – for example, at night – chondrocytes experience a decreased oxygen supply (a status termed “hypoxia”). However when the status changes to an enhanced mobility in the morning, joint SF receives elevated supply of oxygen (a situation termed “re-oxygenation”). Such increased content of oxygen can be, however, deleterious for the homeostasis of the chondrocytes – the cells that in adults lack mitotic activity.

As “normal” SF contains no hyaluronidase activity, it has been inferred that oxygen-derived reactive species/metabolites are involved in a self-perpetuating process of hyaluronan catabolism within the joint (Grootveld *et al*., 1991). This process has been considered responsible for the short – about twelve hours – half-life of native HA macromolecules in SF.

To understand how to keep a radical reaction active/self-perpetuating, its propagation stage should first be analyzed. Let us assume that a peroxyl-type macromolecular radical (AOO·) exists within SF. Taking into account the relatively high reactivity/affinity of the unpaired electron on the oxygen, the following particular reaction step can be assumed

(9) AOO·+ HA → AOOH +A·

where HA abbreviates the hyaluronan, in which H stands for a hydrogen atom – the abstraction of which by the AOO· radical leads to the formation of an A· macroradical. In the case when A· is a carbon macroradical, it is especially this reactant, which traps the excess of dioxygen molecule(s) according to the reaction

(10) $$\text{A}\cdot \text{+}{\text{O}}_{\text{2}}\text{→ AO}\text{O}\cdot$$

Hence, simply by combining the two reactions **9** and **10**, the net reaction

$$\text{HA +}{\text{O}}_{\text{2}}\text{→ AOOH net reaction}$$

corroborates the statement that one particular function of (a high-molar-mass) HA is to trap the oxygen excess during the stage of joint re-oxygenation.

## Participation of biogenic transition metals in physiologic catabolism of hyaluronan

Analogously to the decomposition of lipid hydroperoxides (reactions **6** and **8**) caused by a transition metal, similar reactions could be suggested for decomposition of the cumulating AOOH hydroperoxides

(11) $$\text{AOOH + Fe(II)→ A}\text{O}\cdot \text{+ H}{\text{O}}^{-}\text{+ Fe(III)}$$

(12) $$\text{AOOH + Fe(III)→ AO}\text{O}\cdot \text{+ Fe(II)+}{\text{H}}^{\text{+}}$$

As evident, while the reactant/”propagator” entering into reaction **9** is regenerated/recycled by reaction **12**, reaction **11** produces an alkoxyl macroradical. The ratio between the generated AOO· to AO· radicals is however governed by the present iron, or more precisely by the ratio of Fe(III) to Fe(II). However, in the case of a sufficiently high level of ascorbate, an efficient reductant of ferric cations, actual concentration of Fe(II) ions exceeds that of Fe(III), and thus AO· radicals should prevail. These radicals could, similarly to the AOO· ones, propagate the radical chain reaction as follows

(13) AO·+ HA → AOH +A·

however assuming the quenching of AO· radicals by ascorbate, the products would now contain “oxidized” hyaluronan macromolecules *plus* DHA and/or its hydrolyzates.

Higher reactivity of AO· radicals with ascorbate compared to that of the AOO· radicals, closely correlates with the about 1,000-times higher value of the rate-constant (*k*) in the case of reduction of alkoxyl radicals. For example, the *k* values estimated for the reactions between ascorbate and a tocopheryl (T) radical TO· or peroxyl-type radical TOO·, equal 1.6×10^9^ or (1–2)×10^6^ M^−1^s^−1^, respectively and, hence, the half-life of the alkoxyl radical is much shorter than that of peroxyl – microseconds *vs*. seconds. Analogously, the redox potential of the pair RO·, H^+^/ROH=1.6 V exceeds significantly that of ROO·,H^+^/ROOH=1.0 V.

**Scheme 2:**
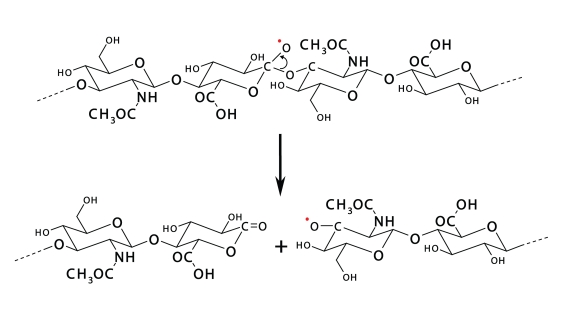
AO· strand scission may be due to β-cleavage of the radical formed at, e.g. C(1) on the ring of D-glucuronate/D-glucuronic acid.

Yet, concerning the radical reactions of carbohydrate polymers, the strand scission of AO· as well as of AOO· (intermediate) macroradicals represents a very plausible pathway, by which polymer fragments of shorter molecular size shall be generated (cf. Scheme 2) (see also Refs. Rychlý *et al*., 2006; Šoltés *et al*., 2006; Stern *et al*., 2007).

Along with the above described hyaluronan fragmentation reactions, the radical attack on the D-glucuronate/D-glucuronic acid and *N*-acetyl-D-glucosamine moieties can also lead to the “opening” of rings without breaking the polymer chain (Kogan *et al*., 2007; 2008; Hawkins and Davies, 1996).

## Actors of physiologic catabolism of hyaluronan in the synovial fluid

The concentration of ascorbate in SF of healthy subjects reaches the values close to those established in blood serum, i.e. 40–140 µM (Wong *et al*., 1981). The contents of transition metals in both body fluids are listed in Table 3.

**Table 3:** Contents of transition metals in blood serum of healthy human volunteers and in *post mortem* collected SF from subjects without evidence of connective tissue disease.

Element	Mean concentration in blood serum [µg/100 mL]^a^	Mean concentration in synovial fluid [µg/100 g]^a^
Manganese	2.4 (0.44)^b^	2.4 (0.44)
Iron	131.7 (23.6)	29.0 (5.19)
Nickel	4.1 (0.70)	1.2 (0.20)
Copper	97.0 (15.3)	27.5 (4.33)
Zinc	115.4 (17.7)	17.6 (2.69)
Molybdenum	3.4 (0.35)	1.0 (0.10)

^a^ Reported by Niedermeier and Griggs (1971).

^b^ Data in parentheses are the values in µM calculated in assumption that 100 g of SF has a volume of 100 mL.

As evident from the data listed in Table 3, iron and copper are the two prevailing redox active transition metals in SF. It should be, however, pointed out that the respective levels of ca. 5.2 and 4.3 µM do not represent those, which are (freely) disposable to catalyze the oxidative catabolism of hyaluronan within SF. As has been reported, the availability of iron to stimulate *in vivo* generation of ·OH radicals is very limited, since concentrations of bleomycin-detectable, i.e. “free” iron, are rarely larger than 3 µM in human samples (Halliwell and Gutteridge, 1990).

**Figure 2:**
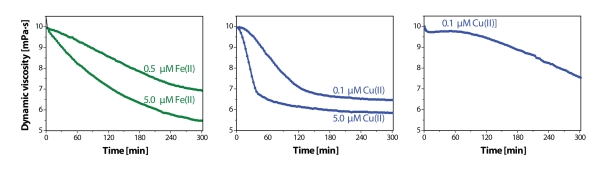
Time dependences of dynamic viscosity of hyaluronan (P9710-2A) sample solutions (2.5 mg/mL). Left panel: Solutions of the HA sample with addition of 100 µM ascorbic acid immediately followed by admixing 0.5 or 5.0 µM of FeCl2. Middle panel: Solutions of the HA sample with addition of 100 µM ascorbic acid immediately followed by admixing 0.1 or 5.0 µM of CuCl2. Right panel: The curve represents an assay, in which 0.1 µM of CuCl2 was added to the HA sample solution 9 minutes before admixing 100 µM ascorbic acid (for details see Šoltés *et al*., 2007).

Figure 2 illustrates the viscosity-time profiles of a model situation, where – along with 100 µM ascorbate – a single transition metal is involved (Valachova *et al*., 2009). As evident, a significant reduction of the solution dynamic viscosity (η), corresponding to the degradation of the high-molar-mass HA sample, clearly indicates a concentration-dependent manner for each metal (cf. left and central panels in Figure 2). As can be seen, the character of the time dependence of η value upon the addition of FeCl_2_ (5.0 µM) can be described as a gradual monotonous decline, while the addition of CuCl_2_ (5.0 µM) resulted in a literally “dramatic” drop of η value within a short time interval (30 min), after which the decrease of the solution dynamic viscosity continued, yet at a much lower rate. A possible explanation of this dissimilarity lies most probably in different reaction kinetics of the processes leading to generation of oxygen-derived reactive species in the system ascorbate *plus* Cu(II) and in that comprising ascorbate *plus* Fe(II), i.e. Weissberger's and Udenfriend's oxidative systems.

A similar drop of η value and two-phase reaction kinetics are identifiable upon the addition of even a minute (0.1 µM) amount of CuCl_2_ (see Figure 2, central panel), however when Cu(II) addition preceded that of ascorbate (right panel), the decline of the solution viscosity was much less marked. A plausible explanation of this observation is that a delay in ascorbate addition (9 min) allowed the Cu(II) ions to reach the “binding sites” within the HA macromolecule. The domain of the coordinate-bound copper formed in such a way seems to be “hindered” and hence less accessible for a direct complexation/interaction with the ascorbate anion, a condition that is required for formation of the Weissberger's oxidative complex. As postulated, hyaluronate binding sites indicate a (weak) selectivity not only toward copper but also to (isoelectronic) zinc cations (Magnani *et al*., 1999). On the contrary, manganous and presumably also (isoelectronic) Fe cations form simple hyaluronate salts with D-glucuronate moieties (Pirc *et al*., 2004). Thus, the four transition metal elements, namely iron, copper, manganese, and zinc, may by their presence/absence create a variety of conditions under which HA oxidative degradation proceeds.

There exists, however, a marked difference between degradation of hyaluronan *in vitro*/*in situ* and its *in vivo* catabolism. Under physiological conditions, the SF viscosity does not indicate any changes since the content of “native” hyaluronan remains constant due to permanent *de novo* production of megadalton HA macromolecules by (stimulated) synoviocytes. Thus, the self-perpetuating oxidative (non-enzymatic) HA catabolism in SF represents a rather delicate and properly balanced mechanism that presumably plays significant role in regulating the physiological – normoxygen – homeostasis for chondrocytes. At the same time, the polymer fragments formed, which are removed by drainage pathways from the joint, serve most likely as chemical messengers/feedback molecules used for adjustment of the optimum mode of functioning of the synovial membrane and of the HA-producing cells, synoviocytes, localized within. In the other words, during physiologic joint functioning, the hyaluronan in SF plays the role of a “scavenger antioxidant”, whereas the produced polymer fragments can subsequently serve as messengers mediating information on the changes occurring in the homeostasis of the joint.

High “protective/scavenging efficiency” of hyaluronan against the action of e.g. ·OH radicals has been earlier pointed out by some authors (Myint *et al*., 1987; Presti and Scott, 1994). Presti and Scott (1994) described that high-molar-mass hyaluronan (1218 kDa) was much more effective than the lower-molar-mass (668 and 176 kDa) HAs in scavenging ·OH radicals generated by a Fenton-type system comprising glucose and glucose oxidase *plus* Fe^2+^-EDTA chelate.

## Pathophysiologic catabolism of hyaluronan in synovial fluid

Despite their exceedingly simple primary structure, the HA macromolecules have extraordinarily wide-ranging and often opposing biological functions. As recently reported by Stern *et al*., the unadorned HA polymer functions size-specifically and its fragments constitute a very information-rich system (Stern *et al*., 2006). While “space-filling” megadalton hyaluronans, synthesized by HA synthases (HAS1 and HAS2) (Itano *et al*., 1999; Weigel and DeAngelis, 2007), are immunosuppressive and anti-angiogenic (McBride and Bard, 1979; Delmage *et al*., 1986; Feinberg and Beebe, 1983), the intermediate-sized HA-polymer fragments are inflammatory, immunostimulatory, and highly angiogenic (Noble, 2002; West *et al*., 1985; Jiang *et al*., 2007).

There are two distinct modes to cleave *in vivo* the extended native HA chains: the enzymatic, involving participation of the hyaluronidases (e.g. Hyal-2) and the chemical one, involving oxygen-derived reactive species (Stern *et al*., 2007). Yet, due to the fact that SF is lacking any hyaluronidases, it is reasonable to assume that it is the latter mechanism, which might be involved also during HA catabolism in the inflamed joint. As can be seen from Figure 2, any transition metal, i.e. iron or copper, can play an active role in the oxidative HA catabolism. However, the increase in Cu(II) concentration within the joint (and particularly in SF) could lead to a really very rapid degradation of the native HA macromolecules.

**Figure 3:**
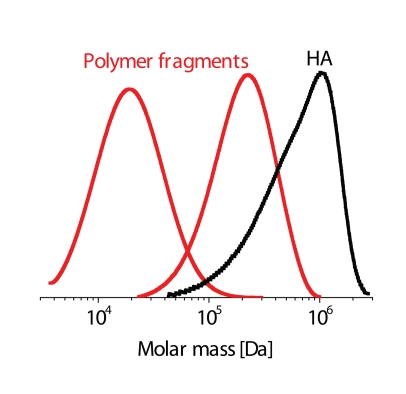
Comparison of MMD of the megadalton HA sample and those degraded in situ by oxygen-derived reactive species (cf. also Figure 2 and Ref. Šoltés *et al*., 2007).

Figure 3 exemplifies the impact of two different oxidative systems comprising Cu(II) ions. As evident, a megadalton HA sample (M_w_ = 1.2 MDa) degrades *in situ* to intermediate-sized polymer fragments, which mean molar masses are reduced by one or even two orders of magnitude. Interestingly, the unimodal and relatively narrow molar-mass distribution (MMD) of the megadalton HA sample (M_w_/M_n_ = 1.8) remains retained even upon cleaving the polymer (compare the three MMD curves in Figure 3).

Some differences should be, however, pointed out between the processes taking place in a joint (or locally within SF) and in a well-stirred reaction mixture under *in situ* conditions. In the bloodstream, the copper atoms are carried by ceruloplasmin, belonging to the so-called “acute phase proteins”, which concentration increases markedly under certain circumstances (Shukla *et al*., 2006). Higher ceruloplasmin concentration in the blood plasma would mean a larger amount of copper that crosses the synovial membrane. Yet, due to the SF gel-like consistency, the copper ions entering into this specific environment should start their “dramatic” oxidative action in the vicinity of the synovial membrane. How efficiently the chemically generated ·OH radicals are “scavenged” within this microenvironment by the locally disposable albumin as well as by the HA polymer fragments of lower molecular size, remains questionable. The oxidative process may escape the control mechanisms and damage/disrupt the synovial membrane. Moreover, the intermediate-sized HA-polymer fragments generated within this microenvironment could participate in the activation of “defender” cells. They may further aggrandize the inflammation state of the injured tissue(s) because the HA-polymer fragments can augment in turn inflammatory responses. As reported by Jiang *et al*., the HA fragments in the e.g. 2×10^5^ Da range induce in macrophages the expression of a number of inflammatory mediators, including chemokines, cytokines, growth factors, proteases, and nitric oxide (Jiang *et al*., 2007). In this way, the oxidants generated by activated defender cells may enlarge the damage within the involved joint tissues such as the synovial membrane.

Moreover, it appears that reactive oxygen species disrupt copper binding to ceruloplasmin, thereby releasing “free” copper ions, which in turn may promote oxidative pathology (Shukla *et al*., 2006). The damage can be manifested by visually localizable cardinal symptoms of inflammation – color, dolor, rubor, tumor, and functiolaesa. Yet, although less distinct, repeated (micro-acute) inflammatory injures may lead to a disastrous outcome – e.g. an autoimmune disease – such as rheumatoid arthritis.

The mutual substitution of (isoelectronic) transition metals, their versatile coordination chemistry and redox properties, along with changes in their compartmentalization and binding to the designated tissue/cell constituents may reflect certain non-specific physiologic responses of the organism to pathologic stimuli (Valko *et al*., 2005). Rheumatoid arthritis can be classified as one of the diseases of particular interest in this respect. In the synovial fluid from patients with rheumatoid arthritis compared to SF from normal/healthy subjects, the mean concentration of copper is increased by a factor of three and that of iron and zinc by 2 and 3.5, respectively (Niedermeier and Griggs, 1971).

There are several chronic (inflammatory) conditions, in which “abnormalities” in the metabolism of trace/transition metal(s) are at present in the focus of biomedical research. The diseases – such as the autoimmune rheumatoid arthritis, the neurodegenerative Alzheimer's and Parkinson's diseases – are classified as socially debilitating. Considering the fact of permanently extending mean life-span of the human population, these diseases create serious ethical/societal issues, like that of euthanasia.
